# Effects of Vitrectomy on Recurrent Macular Edema due to Branch Retinal Vein Occlusion after Intravitreal Injection of Bevacizumab

**DOI:** 10.1155/2013/415974

**Published:** 2013-02-20

**Authors:** Tatsuya Yunoki, Keiichi Mitarai, Shuichiro Yanagisawa, Tsuyoshi Kato, Nobuo Ishida, Atsushi Hayashi

**Affiliations:** ^1^Department of Ophthalmology, Graduate School of Medicine and Pharmaceutical Sciences, University of Toyama, 2630 Sugitani, Toyama 930-0194, Japan; ^2^Division of Ophthalmology, Takaoka Municipal Hospital, 4-1 Takaramachi, Takaoka 933-8550, Japan; ^3^Ishida Eye Clinic, 2-2-31 Honcho, Jouetsu 943-0832, Japan

## Abstract

*Purpose*. To evaluate the effects of pars plana vitrectomy (PPV) on recurrent macular edema due to branch retinal vein occlusion (BRVO) after intravitreal injections of bevacizumab (IVB). *Methods*. This retrospective study included 22 eyes of 22 patients who underwent single or multiple IVB injections for macular edema due to BRVO and showed a recurrence of macular edema. All patients then underwent PPV and were followed up for more than 6 months after the surgery with examinations of best corrected visual acuity (BCVA) and optical coherence tomography (OCT). OCT parameters were central macular thickness (CMT) and average retinal thickness in a 1-mm-diameter circular region at the fovea (MRT). *Results*. Mean BCVA, CRT, and MRT were significantly improved from the baseline after PPV. Greater improvement of BCVA, CRT, and MRT was obtained after 1 month of IVB than after 6 months of PPV. No eyes showed worsening of macular edema after the surgery. *Conclusion*. PPV improved BCVA and recurrent macular edema due to BRVO, but PPV that was less effective than IVB had been in the same patients. PPV may be one of the treatment options for recurrent macular edema due to BRVO after IVB.

## 1. Introduction

Macular edema due to branch retinal vein occlusion (BRVO) is a major cause of visual loss. Macular grid laser photocoagulation, intravitreal injections of steroids, and vitrectomy have been attempted to treat the macular edema secondary to BRVO [1–4]. Recently, we and others have reported the effectiveness of intravitreal injections of bevacizumab (IVB) for macular edema secondary to BRVO [5–8]. IVB for macular edema secondary to BRVO is an effective treatment for the short term, providing immediate improvement of visual acuity and macular edema. However, macular edema due to BRVO seems to recur frequently in spite of multiple IVB [5–8]. There are no effective treatments for recurrent macular edema secondary to BRVO. 

Several groups have reported the effectiveness of vitrectomy surgery for macular edema due to BRVO [1, 4, 9]. The mechanisms of resolution of macular edema by vitrectomy have not been clarified yet. Using optical coherence tomography (OCT), we examined the effects of pars plana vitrectomy (PPV) for recurrent macular edema due to BRVO after multiple IVB with or without apparent vitreomacular traction and/or an epiretinal membrane. 

## 2. Patients and Methods

### 2.1. Patients

We retrospectively examined 22 eyes of 22 consecutive patients (18 female and 4 male, average 72.1 ± 6.3 years old) with macular edema due to BRVO at Toyama University Hospital and Ishida Eye Clinic from February 2008 to March 2011. The macular edema was confirmed by OCT, which revealed in all cases a central macular thickness (CMT) of more than 250 *μ*m. Patients who had BRVO without involvement of macular edema or without a decrease in visual acuity were excluded. 

The research was conducted in accordance with the Institutional Guidelines of University of Toyama and was approved by the Institutional Review Board. The procedures conformed to the tenets of the World Medical Association's Declaration of Helsinki. Written informed consent was obtained from each of the patients after they were provided sufficient information about the procedures.

### 2.2. Primary Therapy of Intravitreal Injection of Bevacizumab

All patients in this study underwent an intravitreal injection of bevacizumab (IVB) as a primary treatment at their first visit to Toyama University Hospital. The intravitreal injection of bevacizumab was performed as follows. After topical anesthesia with 2% lidocaine was applied, the eye was irrigated with 10% povidone iodine. Then, from 0.1 to 0.2 mL of  2% lidocaine was injected into the subconjunctival space around the anticipated injection site. At 3.5 to 4 mm from the limbus, 1.25 mg (50 *μ*L) of bevacizumab was injected into the vitreous cavity with a 29-gauge needle. Antibiotic eye ointment was applied to the cul-de-sac at the end of the injection. An eye drop of antibiotic was given daily for several days after the IVB.

### 2.3. Followup Examination and Treatment

 After the first IVB, all patients were followed every 4 weeks with best-corrected visual acuity examinations, ophthalmic examinations, and OCT examinations (RTVue-100; Optovue Inc, Fremont, CA, USA). The OCT examinations included measurements of central retinal thickness (CRT) and average retinal thickness in a 1 mm-diameter circular region at the fovea (MRT). CRT was measured manually in the OCT images. MRTs were obtained using the EMM5 software for the RTVue-100. 

The decimal visual acuity was converted to units of the logarithm of the minimum angle of resolution (logMAR) for use in statistical analyses.

When a recurrence of macular edema was detected by a more than 20% increase in CRT compared to that in the previous examination and subjective symptoms deteriorated, another intravitreal injection of bevacizumab (1.25 mg) was given in the same manner. We continued IVB therapy for recurrent macular edema due to BRVO. When the macular edema recurred after multiple IVB treatments, we performed PPV as the next treatment for the recurrent macular edema, irrespective of the presence of vitreous adhesion and/or an epiretinal membrane by OCT. The surgery was performed after an informed consent was obtained.

All patients in the study underwent a standard 25 gauge three-port pars plana vitrectomy (PPV). During vitrectomy, posterior vitreous detachment was created and confirmed with particles of triamcinolone acetonide, then the inner limiting membrane (ILM) was peeled. The area of ILM peeling was about 2.5–3.0 disc diameters around the fovea. During the PPV, laser photocoagulation was performed on the avascular area of BRVO. For all phakic eyes, phacoemulsification and intraocular lens implantation were performed. 

After the PPV, all patients were followed every 4 weeks for at least 6 months with best corrected visual acuity (BCVA), ophthalmoscopic examinations, and OCT examinations (CRT and MRT). We provided no additional treatments for residual macular edema after PPV. 

We divided the 22 eyes into two groups according to whether vitreomacular traction and/or an epiretinal membrane was observed by OCT before the PPV. The group of the eyes with apparent vitreo-macular traction included 9 eyes and that without apparent vitreo-macular traction included 13 eyes. We compared postoperative changes in BCVA and in morphologic parameters of OCT between the two groups.

### 2.4. Statistical Analyses

We analyzed the data at baseline, 1 month after first IVB, just before PPV, and 1 month, 3 months, and 6 months after PPV. All statistical analyses were performed with JMP 9 (SAS Institute, Cary, NC, USA). Comparisons between the two groups were done by analysis of variance (ANOVA). *P*  values less than 0.05 were considered to indicate statistical significance. 

## 3. Results

### 3.1. Baseline Characteristics and IVB Treatment

The baseline characteristics of patients are shown in Table 1. The mean age of the patients (4 men, 18 women) was 72.1 ± 6.1 years (mean ± standard deviation, range 61–84 years). The mean BCVA at baseline was 0.66 ± 0.37 logMAR units (range 0.55–1.22). The parameters of OCT at baseline were 474 ± 143 *μ*m for mean CRT (range 261–886), 559 ± 131 *μ*m for mean MRT (range 333–745). 

All 22 eyes received IVB at the first visit, at 1 month after the first IVB, and both BCVA and OCT parameters (CRT and MRT) significantly improved from the baseline to 0.28 ± 0.31 (*P* < 0.05), 261 ± 73 *μ*m (*P* < 0.01), and 325 ± 51 *μ*m (*P* < 0.05), respectively (Table 1). 

The mean number of IVB replications was 2.45 ± 1.56 (range 1–7) at the time of preoperation. The mean interval of multiple IVBs was 81 ± 53 days (range 34–280). 

### 3.2. Preoperative Characteristics

The mean BCVA at preoperation was 0.55 ± 0.36 logMAR units (range 0–1.1) (Table 1). The parameters of OCT at preoperation were 444 ± 160 *μ*m for mean CRT (range 251–700) and 470 ± 174 *μ*m for mean MRT (range 210–820) (Table 1). 

 Because of recurrent macular edema, all 22 patients underwent vitrectomy surgery, with a mean duration of 248 ± 117 days (range 60–387) after the initial IVB. Of the 22 eyes, 18 (82%) were phakic and underwent cataract surgery at the same time. Apparent epiretinal membrane and/or vitreomacular traction was observed by OCT in 9 eyes (41%). The other 13 eyes (59%) showed a recurrent macular edema without apparent vitreomacular traction. No postoperative complications were observed in this study. 

### 3.3. Mean Visual Acuity

**Figure 1:**
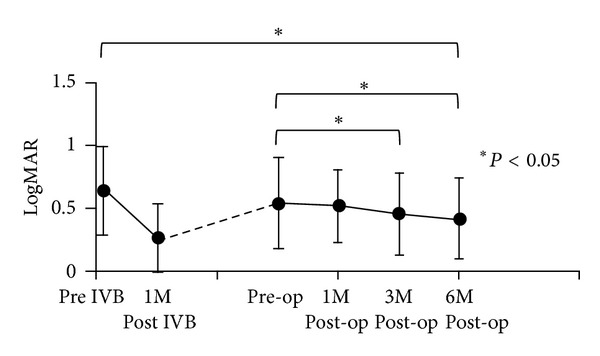
Changes in mean best corrected visual acuity. Mean BCVA was significantly improved after 1 month of IVB compared to the baseline. Mean BCVA was significantly improved at 3 months and 6 months after vitrectomy, compared to the preoperative mean BCVA (*P < 0.05). BCVA: best corrected visual acuity; IVB: intravitreal injection of bevacizumab; post IVB: 1 month after IVB.

The changes in mean BCVA of all 22 eyes are shown in Figure 1. The mean BCVA significantly improved from 0.66 ± 0.37 logMAR units to 0.28 ± 0.31 at 1 month after IVB (*P* < 0.05). However, the mean BCVA worsened to 0.55 ± 0.36 before the PPV because of recurrent macular edema. After the PPV, the mean BCVA improved to 0.52 ± 0.29 at 1 month, 0.46 ± 0.32 at 3 months (*P* < 0.05), and 0.43 ± 0.33 at 6 months (*P* < 0.05). At 3 and 6 months after the PPV, the mean BCVA was significantly better than the pre-operative mean BCVA. However, greater improvement of BCVA was obtained at 1 month after IVB than at 6 months after PPV. 

Improvement or deterioration of BCVA at 6 months after the PPV was defined by changes of more than 0.3 logMAR units from the preoperation values. Changes less than 0.3 logMAR units were considered to indicate maintained status. At 6 months after the PPV, BCVA improved in 8 eyes (36%), was maintained in 13 eyes (60%), and deteriorated in 1 eye (4%). 

### 3.4. Morphologic Parameters

#### 3.4.1. CRT

**Figure 2:**
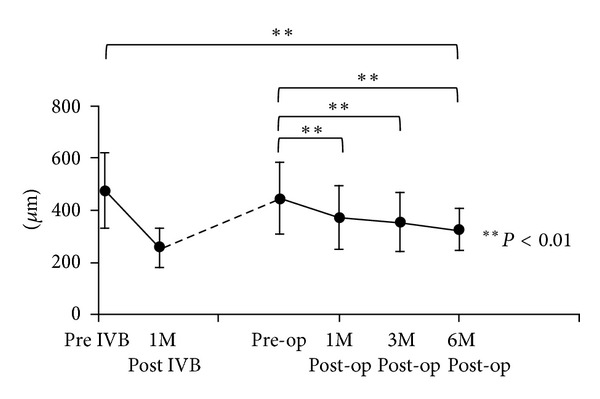
Changes in mean central retinal thickness. Mean CRT was significantly improved after 1 month of IVB compared to the baseline. Mean CRT was significantly improved at 1 month after vitrectomy, compared to the pre-op CRT (**P < 0.01). CRT: central retinal thickness; post IVB: 1 month from IVB.

As shown in Figure 2, the mean CRT of 22 eyes significantly improved from 480 ± 144 *μ*m at baseline to 261 ± 73 *μ*m at 1 month after IVB (*P* < 0.01). However, the mean CRT returned to 452 ± 142 *μ*m before PPV with no significant difference from the baseline due to recurrent macular edema. The mean CRT significantly improved to 379 ± 122 *μ*m (*P* < 0.01) at 1 month, 355 ± 110 *μ*m at 3 months (*P* < 0.01), and 327 ± 78 *μ*m at 6 months after the PPV (*P* < 0.01). However, greater improvement of CRT was obtained at 1 month after IVB than at 6 months after PPV. No eyes showed worsening of CRT after the surgery.

#### 3.4.2. MRT

**Figure 3:**
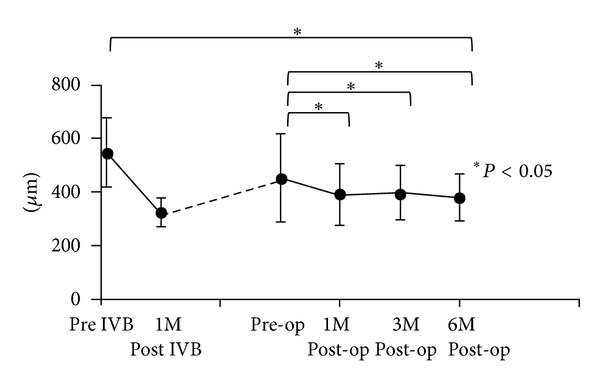
Changes in average mean retinal thickness. Mean MRT was significantly improved after 1 month of IVB compared to the baseline. Mean MRT was significantly improved at 1 month after vitrectomy, compared to the pre-op MRT (*P < 0.05). MRT: mean retinal thickness; post IVB: 1 month from IVB.

 As shown in Figure 3, the mean MRT of 22 eyes significantly improved from 559 ± 131 *μ*m at baseline to 325 ± 51 *μ*m at 1 month after IVB (*P* < 0.05). However, the mean MRT returned to 468 ± 177 *μ*m before PPV with no significant difference from the baseline due to recurrent macular edema. The mean MRT significantly improved to 394 ± 113 *μ*m (*P* < 0.05) at 1 month, 397 ± 99 *μ*m at 3 months (*P* < 0.05), and 378 ± 88 *μ*m at 6 months after the PPV (*P* < 0.05). However, greater improvement of MRT was obtained at 1 month after IVB than at 6 months after PPV. No eyes showed worsening of MRT after the surgery.

### 3.5. Effects of Apparent Vitreomacular Traction

**Figure 4:**
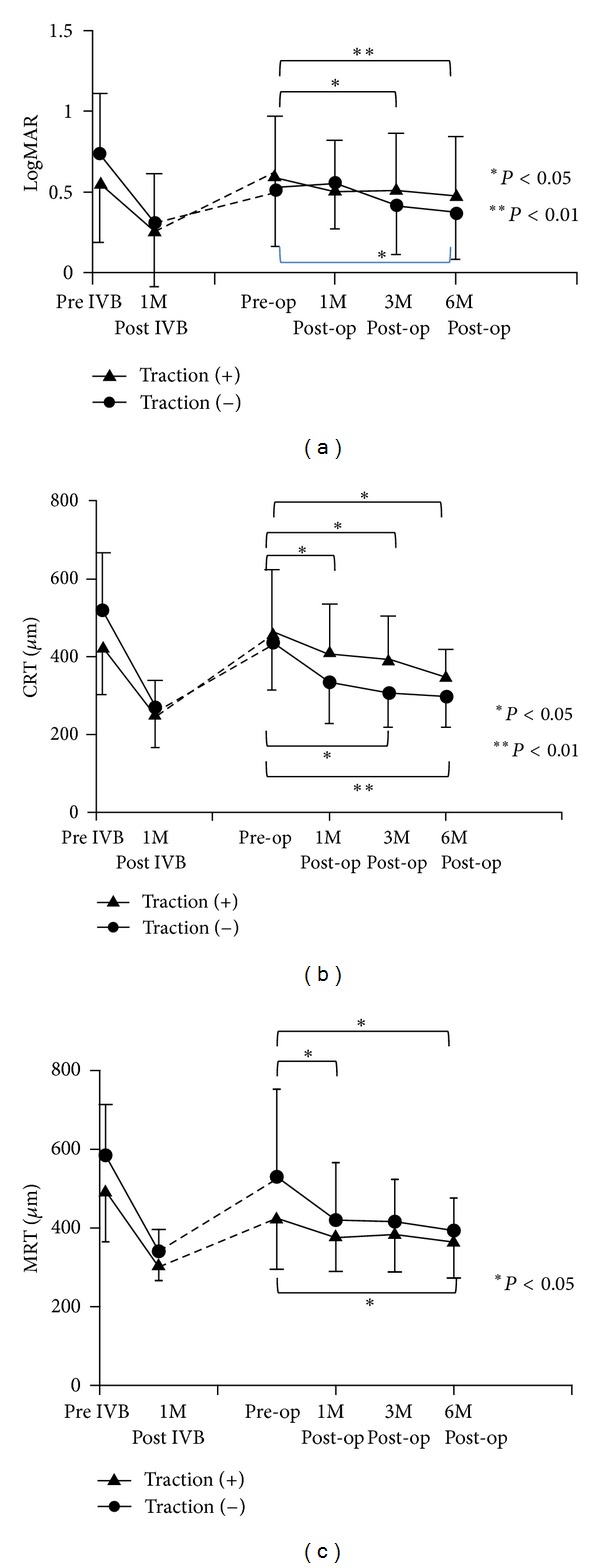
Changes in BCVA (a), CRT (b), and MRT (c) of two groups: one group of the eyes with apparent vitreomacular traction and/or an epiretinal membrane and the other group of the eyes without apparent vitreomacular traction. (a) Mean BCVA was significantly improved in both groups after vitrectomy (*P < 0.05, **P < 0.01). There was no significant difference between the two groups at any time point. (b) Mean CRT was significantly improved in both groups after vitrectomy (*P < 0.05, **P < 0.01). There was no significant difference between the two groups at any time point. (c) Mean MRT was significantly improved in both groups after vitrectomy (*P < 0.05). There was no significant difference between the two groups at any time point.

As shown in Figure 4(a), the mean BCVA significantly improved up to 6 months after the PPV in both groups. The mean BCVA was not significantly different between the two groups at any time point. 

As shown in Figures 4(b) and 4(c), both the mean CRT and mean MRT significantly improved in these two groups until 6 months after PPV was performed; however, mean CRT and mean MRT were not significantly different between the two groups at any time point.No increase in macular edema was observed after PPV in the 22 eyes.

## 4. Discussion

 It is well known that VEGF increases vascular permeability and is associated with macular edema due to BRVO [10]. We and others have shown that anti-VEGF therapy is effective for macular edema due to BRVO [6–8]. However, because IVB therapy, which is an off-label use, is generally required several times due to recurrent macular edema, and because repeating IVB carries small risks of severe complications such as endophthalmitis, retinal detachment, or cerebral infarction [11, 12], there is a need for other effective treatments for recurrent macular edema due to BRVO after multiple IVB. 

 In this study, we treated all patients with IVB at the first visit when the visual acuity was decreased, and macular edema involving more than 250 *μ*m of the fovea was detected because we still do not have enough information to distinguish between spontaneously resolving macular edema and persistent macular edema in BRVO. Since persistent macular edema is a major cause of visual acuity loss in BRVO [2, 13, 14], it is important to treat macular edema due to BRVO in the early phase.

We employed PPV as the next treatment modality for recurrent macular edema when macular edema recurred after one or more IVB treatments, because PPV has been shown to be effective for diabetic macular edema with or without a thickened posterior hyaloid [15–17]. Bertelmann et al. showed that the posterior vitreous cortex in patients with a history of BRVO attached more frequently to the retina, compared with healthy age-matched controls [18]. We did not detect apparent vitreomacular traction by ophthalmoscopy and OCT at the first visit in this series of patients. However, we confirmed vitreomacular traction and/or an epiretinal membrane in 9 eyes (41%) by OCT after a couple of IVB rounds. The mechanisms of resolution of macular edema after a vitrectomy surgery have not been clarified yet, but several mechanisms have been proposed. One of them is the direct removal of vitreous traction on the macula by removing the attached vitreous and epiretinal membrane [16]. Other mechanisms are improvement of the oxygenation in the ischemic retina and an increased clearance of inflammatory factors from the retinal surface by removing the vitreous gel [1, 9, 15, 17]. Our results showed that the improving effects of PPV on BCVA, CRT, and MRT were less than those that IVB had shown in the same patients, although the simultaneous cataract surgery might contribute to the improvement of visual acuity after PPV.

We performed ILM peeling in all patients during the PPV. Several authors reported that ILM peeling was beneficial for decompression of macular edema due to BRVO [19–21]. In this study, there were no significant differences in mean BCVA, mean CRT, or mean MRT between the two groups after the PPV, irrespective of the presence of an epiretinal membrane and/or vitreomacular traction. We speculated that detaching posterior vitreous and removing the ILM at the macula might be important for decompression of recurrent macular edema in BRVO. 

 Kriechabaum et al. showed that MRT responded slowly and less impressively after IVB administration for macular edema due to BRVO in comparison with CRT because the MRT reflects a larger retinal area than CRT [22]. In this study, we showed that PPV significantly improved both CRT and MRT at 1 month and after. Although the recovery of CRT and MRT after PPV was slower than that after IVB, we expected that PPV resulted in long-lasting effects on improvement of visual acuity and macular edema in contrast to IVB. 

## 5. Conclusion

 We retrospectively examined the effects of PPV on recurrent macular edema due to BRVO after IVB had been performed in the same patients. Although PPV similarly improved BCVA and macular edema due to BRVO, PPV seemed less effective than IVB and required more time to improve the BCVA and macular edema. However, no increase in macular edema was observed after PPV. PPV may be one of the treatment options for recurrent macular edema due to BRVO, irrespective of apparent vitreomacular traction by OCT. 

## Figures and Tables

**Table 1:** Summary of BCVA, CRT, and MRT in the study course.

	Baseline	1 M after IVB	Pre-op	6 M post-op
BCVA (logMAR)	0.66 ± 0.37	0.28 ± 0.31*	0.55 ± 0.36	0.43 ± 0.33*
CRT (*μ*m)	474 ± 143	261 ± 73^#^	444 ± 160	327 ± 78^#^
MRT (*μ*m)	559 ± 131	325 ± 51*	470 ± 174	378 ± 88*

Data are expressed as means ± standard deviations; ^#^ *P* < 0.01; *P < 0.05.

BCVA: best corrected visual acuity; CRT: central retinal thickness; IVB: intravitreal injection of bevacizumab; MRT: mean retinal thickness.
